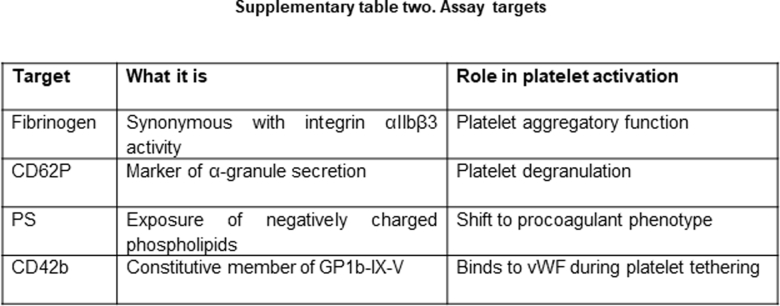# Preanalytical conditions for multiparameter platelet flow cytometry

**DOI:** 10.1016/j.rpth.2023.102205

**Published:** 2023-09-20

**Authors:** Matthew S. Hindle, Lih T. Cheah, Daisie M. Yates, Khalid M. Naseem

**Affiliations:** 1 Discovery and Translational Science Department, Leeds Institute of Cardiovascular & Metabolic Medicine, University of Leeds, UK; 2 Centre for Biomedical Science Research, School of Health, Leeds Beckett University, UK

**Keywords:** anticoagulants, blood platelets, cytofluorometry, flow, flow cytometry, platelet activation, platelet function tests

## Abstract

**Background:**

Flow cytometry is an important technique for understanding multiple aspects of blood platelet biology. Despite the widespread use of the platform for assessing platelet function, the optimization and careful consideration of preanalytical conditions, sample processing techniques, and data analysis strategies should be regularly assessed. When set up and designed with optimal conditions, it can ensure the acquisition of robust and reproducible flow cytometry data. However, these parameters are rarely described despite their importance.

**Objectives:**

We aimed to characterize the effects of several preanalytical variables on the analysis of blood platelets by multiparameter fluorescent flow cytometry.

**Methods:**

We assessed anticoagulant choice, sample material, sample processing, and storage times on 4 distinct and commonly used markers of platelet activation, including fibrinogen binding, expression of CD62P and CD42b, and phosphatidylserine exposure.

**Results:**

The use of suboptimal conditions led to increases in basal platelet activity and reduced sensitivities to stimulation; however, the use of optimal conditions protected the platelets from artifactual stimulation and preserved basal activity and sensitivity to activation.

**Conclusion:**

The optimal preanalytical conditions identified here for the measurement of platelet phenotype by flow cytometry suggest a framework for future development of multiparameter platelet assays for high-quality data sets and advanced analysis.

## Essentials

• Flow cytometry is an excellent technique for measuring blood platelet function.
• Quality multiparameter platelet flow cytometry requires optimized preanalytical conditions.
• Several preanalytical conditions were compared, and the best were determined.
• Using optimal conditions, robust blood platelet flow cytometry can be performed.

## 1 Introduction

The use of fluorescent flow cytometry (FFC) for single-cell analysis is standard in many research settings [1,2], including blood platelet biology. The increased availability of multiparameter FFC, spectral FFC, and cytometry by time-of-flight (CyTOF) has driven a significant rise in the number of parameters that can be measured simultaneously. Accordingly, there has been a parallel expansion and development of multidimensional analytical tools such as t-stochastic neighborhood embedded (tSNE) [3], spanning-tree progression analysis of density-normalized events (SPADE) [4], and flow self-organizing maps (flowSOM) [5]. Concurrent with advances in the wider field of single-cell analysis, platelet flow cytometry has seen various innovations including multidimensional FFC analysis [[6], [7], [8]], phosphoflow and barcoding [9], application of spectral FFC [10] and CyTOF with multidimensional analysis [[11], [12], [13]]. While the latter may show comparable efficacy to FFC [14] and the design of CyTOF assays has been recently described in full [15], it remains relatively inaccessible when compared with FFC, which is common in research and medical institutes worldwide. The continued use of FFC to analyze platelet function both for basic research and clinical use has led to a recent call by the International Society on Thrombosis and Haemostasis Scientific and Standardization Committee (ISTH SSC) on platelet physiology for further studies to facilitate the continued standardization of platelet FFC protocols [16].

We previously designed a multiparameter FFC panel to measure multiple and diverse facets of platelet function to enable the identification of platelet subpopulations in whole blood [6]. We used the same panel in this study to measure (1) fibrinogen binding as an indirect measure of integrin αIIbβ3 activity, (2) P-selectin (CD62P) expression as a marker of α-granule secretion that is ideal for assessing spontaneous activation since its expression is irreversible *in vitro* [17], (3) annexin V binding as a marker of phosphatidylserine (PS) exposure which is representative of platelet procoagulant activity [18,19], and (4) CD42b (GPIb) to identify *bona fide* platelets in the sample as a component of the constitutively expressed GPIb-IX-V complex. CD42b was also considered an additional marker of activation since it may be shed and/or internalized on activation [[20], [21], [22]]. These markers are ideal for assessing the impact of preanalytical conditions as they cover a wide-range of key platelet functions, which are reviewed in detail elsewhere [23]. In order to comment on the effects of the preanalytical conditions, we require robust exposure and expression of all markers analyzed. In these experiments, we used a combination of agonists which we have previously used to induce platelet activation [6].

Beyond assay design and data analysis, which are covered in greater detail elsewhere by general flow cytometry guidelines [1], the optimization of preanalytical conditions is unique to the samples under investigation [24]. Here, we consider the impact of preanalytical conditions on platelet phenotype and function. As platelets are primary cells which are collected as whole blood and require donation on the day of experimental analysis, some preanalytical conditions are especially relevant. These include anticoagulant choice, speed of sample processing, biological sample type, and sample storage. The impacts of these 4 conditions were compared using our 4-parameter FFC assay, and optimal conditions are recommended based on the results. These optimal conditions were then examined for subpopulation analysis by opt-FIt-SNE and Phenograph. The recommendations of this study are intended for researchers aiming to utilize FFC to measure platelet function through the expression of one or more markers and to facilitate identification of blood platelet subsets.

## 2 Methods

### 2.1 Flow cytometry

Samples were run on a Beckman Coulter CytoFLEX RUO Flow Cytometer with 2 lasers (488 nm 50 mW and 638 nm 50 mW) and 4 detectors (525/40 BP, 585/42 BP, 660/10 BP, and 712/25 BP). Avalanche photodiode detector gains were optimized with a LED pulser (quantiFlash, APE) [25]. Automatic compensation was performed with VersaComp Antibody Capture Beads and CytExpert (v2.1).

**Supplementary Figure 1:**
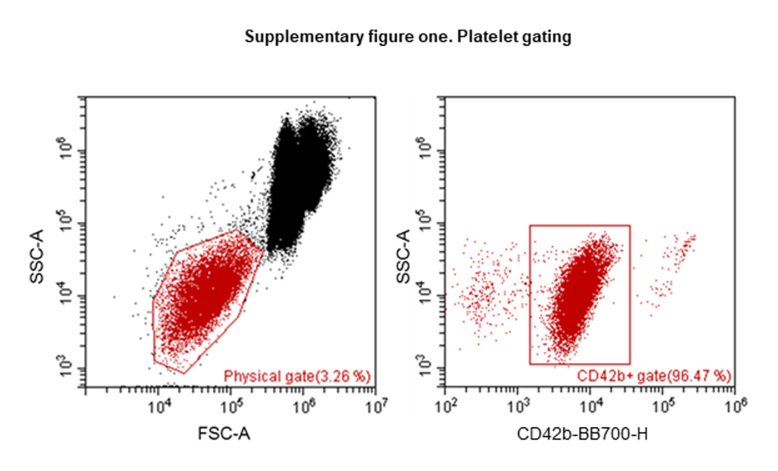


**Supplementary Figure 2:**
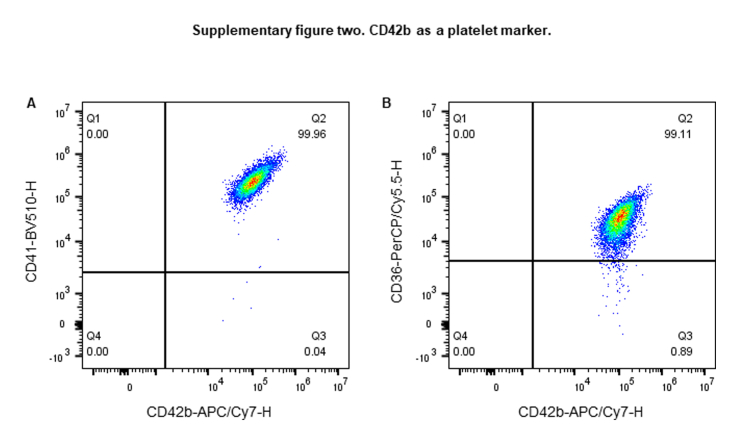


For sample preparation, whole blood, platelet rich plasma (PRP), and washed platelets (WP) were supplemented with calcium (1.8 mM) and Gly-Pro-Arg-Pro (GPRP: 500 μM) to facilitate annexin V binding [19] and prevent fibrin polymerization, respectively [26]. Isolation of PRP and WP is described in the Supplementary Material. Samples were incubated with agonists and antibodies for 20 minutes before fixation with paraformaldehyde solution (0.9% final v/v) [27]. The combination of 2 agonists for protease-activated receptor-1 and GPVI, the peptide agonist SFLLRN (20 μM) and CRP-XL (10 μg/mL), respectively, were used to activate platelets and induce PS exposure [8]. Ten thousand genuine platelet events were recorded based on CD42b expression (Supplementary Figure S1), and doublet events were excluded as previously described [6]. We validated CD42b as a genuine platelet marker (≥99%) by comparing it with CD41 and CD36 expression (Supplementary Figure S2). Positive gates were set with perfectly matched isotype controls (IgG-PE, CD62P) and internal negative controls (EDTA 10 mM, antifibrinogen/annexin V). Antibodies were used at optimal titers (Supplementary Table S1), and assays were designed in accordance with the established guidelines [23,[28], [29], [30], [31]].

### 2.2 Data analysis

Flow cytometry standard (FCS) files were analyzed on CytExpert (v2.1, Beckman Coulter) and FlowJo (v10.7.1, BD Biosciences), and statistical analysis was performed by ordinary one-way analysis of variance with multiple comparisons (Šídák's) on GraphPad Prism (v8.0.0). Statistical significance was accepted as ∗ *P* < .05, ∗∗ *P* < .01, ∗∗∗ *P* < .005, and ∗∗∗∗ *P* < .001. CytExpert was used to export compensated mean/median fluoresence intensities (MFI) and percentage positive data while FlowJo was used to present concatenated FCS data as stacked histograms. Median fluorescence intensities (FIs) were used for all markers except annexin V, which was expressed as the mean FI. In addition to mean ± SD, we also included individual data points. Results were reported as both percent positive and MFI, rather than only presenting a portion of the data acquired. Percent positive is traditional for heterogeneous markers [32] but requires a background control (eg, fluorescence minus one or isotype control), and lacks relevance when all cells are positive. However, MFI does not require gating controls and can differentiate between low and high levels of expression when all cells are positive (ie, 100%). FlowJo was also used to implement opt-FIt-SNE [33,34] and Phenograph (v3.0) [35]. Opt-FIt-SNE was performed with a perplexity of 30, 1000 iterations, and an ANNOY library while Phenograph was performed with 100 nearest neighbors, and data were visualized using ClusterExplorer (v1.6.5). These analyses were performed on CD42b^+^ events from concatenated files of 3 donors.

See Supplementary Materials for venipuncture, and additional methods.

## 3 Results

### 3.1 Anticoagulants

Anticoagulants are perhaps the most important consideration for a platelet biologist, as by their design they can significantly alter and affect platelet function. Here we tested platelet phenotype in 3 commonly used anticoagulants; sodium citrate (BD), sodium heparin (BD), and K_2_EDTA (Greiner Bio-One), all of which were commercially prepared in evacuated blood collection tubes [36].

**Figure 1:**
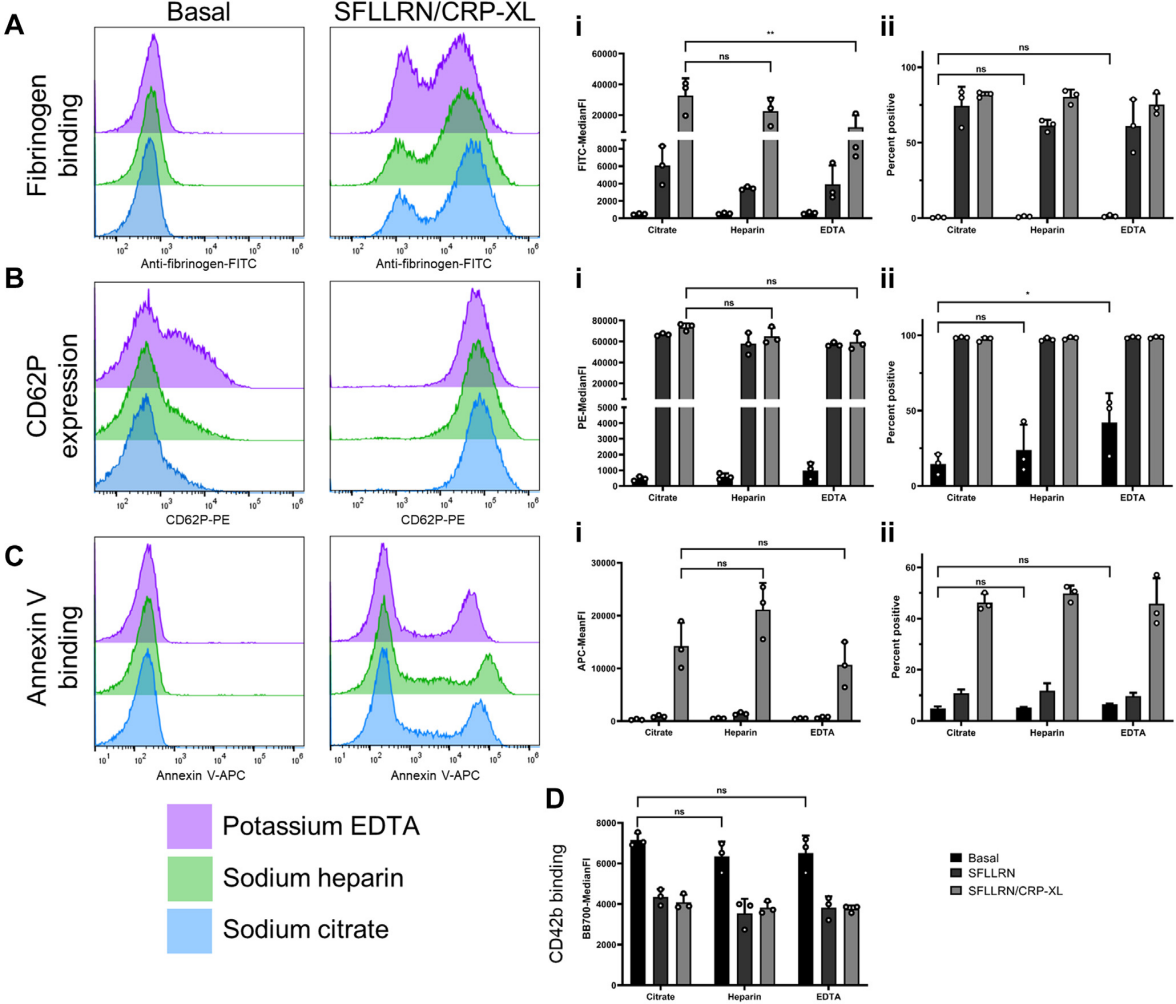
Effects of different anticoagulants on platelet activation. Whole blood drawn into sodium citrate, sodium heparin, and potassium EDTA vacutainers were compared for multiparameter platelet activation at basal or stimulated with SFLLRN (20 μM) alone and with CRP-XL (10 μg/mL). (A) Fibrinogen binding, (B) CD62P expression, (C) PS exposure, (D) CD42b expression, (i) indicates MFI data and (ii) percentage positive data (*n* = 3 ± SD). MFI, mean/median fluoresence intensities.

Measuring fibrinogen binding, we showed that none of the anticoagulants increased basal binding of fibrinogen to the platelet surface (Figure 1A). However, in response to stimulation (SFLLRN/CRP-XL), the greatest binding of fibrinogen (MFI) was observed in citrated blood followed by heparin with a significant reduction in fluorescence when anticoagulated by EDTA (*P* < .01) (Figure 1A). The anticoagulant effect of EDTA is primarily through the chelation of calcium, where platelet–fibrinogen interactions are dependent on the availability of free calcium. When CD62P expression was assessed, we found that the α-granule marker was elevated on the cell surface in heparin (23.8% ± 16.8) and EDTA (*P* < .05, 42% ± 19.5) anticoagulated blood under basal conditions when compared to citrate (14.5% ± 6.7, Figure 1B). CD62P expression in response to stimulation (SFLLRN/CRP-XL) was no different between any of the anticoagulants (Figure 1B). Annexin V binding as a marker of procoagulant platelets showed no differences at basal when comparing the anticoagulants; however in heparinized blood, this showed the highest fluorescence intensity in response to agonists (SFLLRN/CRP-XL), nevertheless this was not significantly increased over citrate (Figure 1C). The procoagulant platelet subpopulation has been described previously as up to two thirds of the total population of platelets [15]. When procoagulant platelets are measured, the proportion of PS positive cells does not change significantly between the anticoagulants and sits within that range (Figure 1C). As a final measure of activity, we recorded the loss of CD42b from the platelet surface, this was shown to be independent of the anticoagulant used at both basal and in the presence of stimuli (SFLLRN/CRP-XL) (Figure 1D).

### 3.2 Sample processing

To understand the effects of short-term storage of whole blood on platelet function, we compared freshly collected whole blood (citrate anticoagulated) with the same sample stored for 1.5 or 4.5 hours at room temperature [37] and compared functional platelet phenotypes.

**Figure 2:**
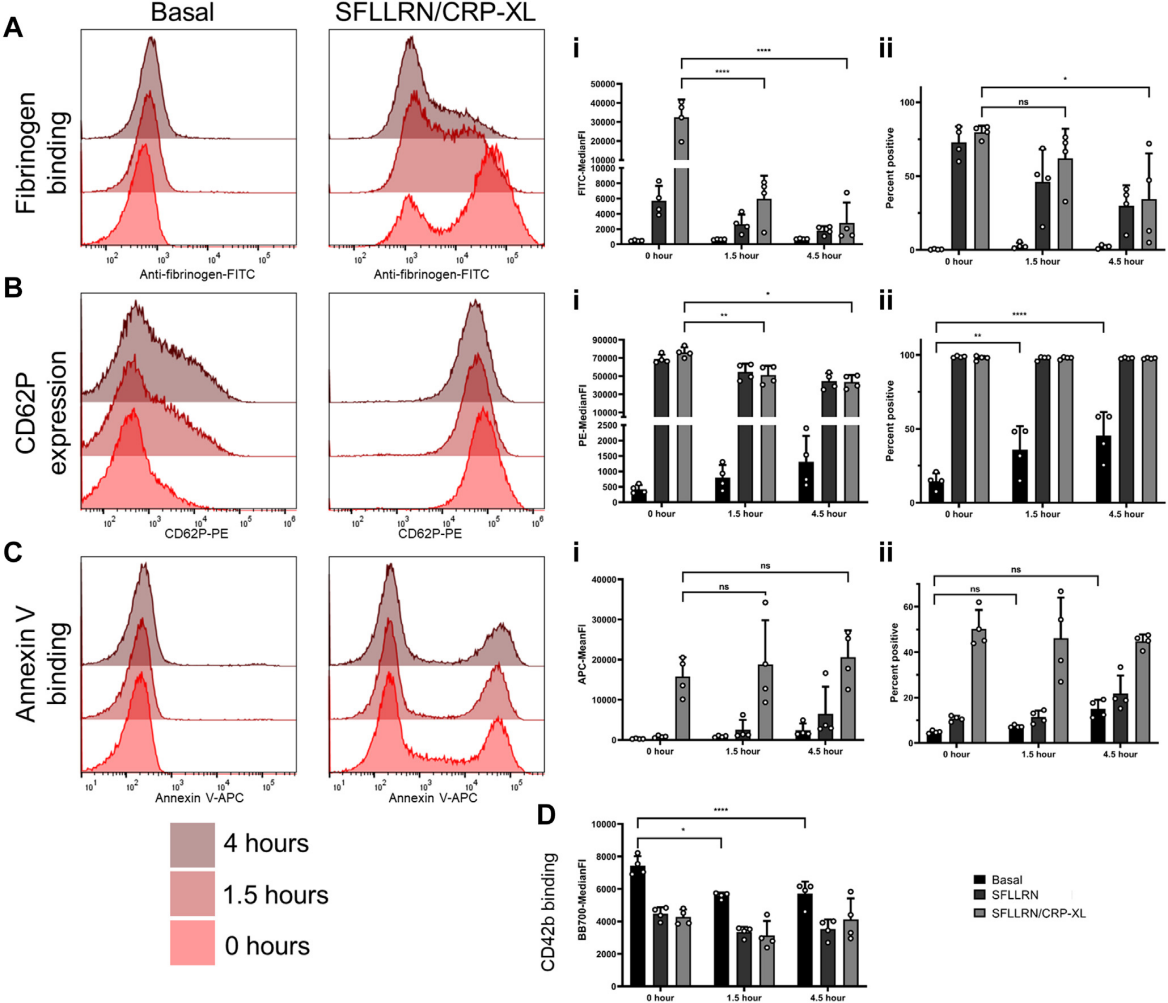
The impact of sample processing time on platelet activity. Multiparameter platelet activation was assayed in whole blood drawn into sodium citrate at 0 hours (freshly drawn), 1.5 and 4.5 hours postdraw at basal or stimulated with SFLLRN (20 μM) alone and with CRP-XL (10 μg/mL). (A) Fibrinogen binding, (B) CD62P expression, (C) PS exposure, (D) CD42b expression, (i) indicates MFI data and (ii) percentage positive data (*n* = 4 ± SD). MFI, mean/median fluoresence intensities.

**Supplementary Figure 3:**
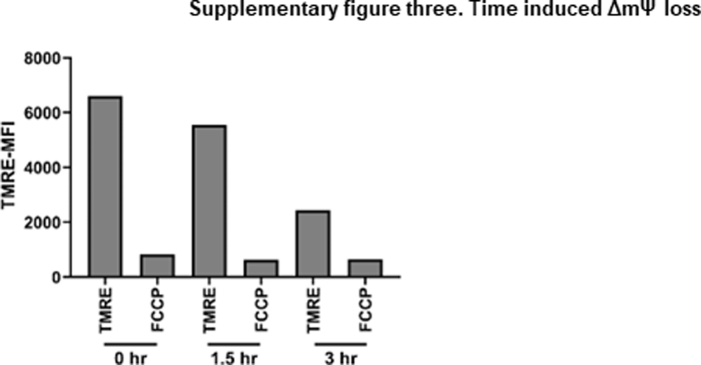


Basal fibrinogen binding did not change over time; however, it showed a dramatic reduction in signal in response to stimulation (SFLLRN/CRP-XL) over the time course tested. MFI was significantly lower at 1.5 hours (*P* < .001), and lowest at 4.5 hours (*P* < .001) (Figure 2A). This reduction was also observed in percent positive cells, where the positive population decreased over time and was significantly reduced at 4.5 hours (*P* < .05) (Figure 2A). Delays in sample processing induced significant artifactual activation, as the percentage expression of basal CD62P increased significantly at 1.5 hours (*P* < .01) and 4.5 hours (*P* < .001), ultimately resulting in near doubling of baseline expression (Figure 2B). Mirroring fibrinogen binding, capacity for CD62P expression by agonist (SFLLRN/CRP-XL) was also diminished at both 1.5 hours (*P* < .01) and 4.5 hours (*P* < .05) (Figure 2B). Annexin V binding was less sensitive to the effect of storage with no significant changes over time (Figure 2C), although there was a trend toward increased basal PS exposure (Figure 2C). We postulate that over a longer period platelet function may continue to decline and basal PS exposure would likely increase [[38], [39], [40]] as PS exposure is also indicative of apoptosis. In support of this observation, mitochondrial membrane potential (ΔmΨ) decreases over a similar time course (Supplementary Figure S3), and a loss of ΔmΨ is typically paired with an increase in PS exposure [6]. The period of storage results in loss of basal CD42b at 1.5 hours (*P* < .05) and 4.5 hours (*P* < .001) (Figure 2D), suggesting spontaneous shedding/and or internalization. However, CD42b expression remained sensitive to agonist-stimulated loss with no difference across the different time points.

### 3.3 Biological samples

Here, we compare citrated whole blood, PRP (isolated from citrate anticoagulated blood), and WP (isolated from acid-citrate dextrose anticoagulated blood) to determine the impact of sample material on the measurement of functional platelet parameters. All samples were paired to allow for accurate comparisons, ie, whole blood, PRP, and WP were all from the same venipuncture from the same donor.

**Figure 3:**
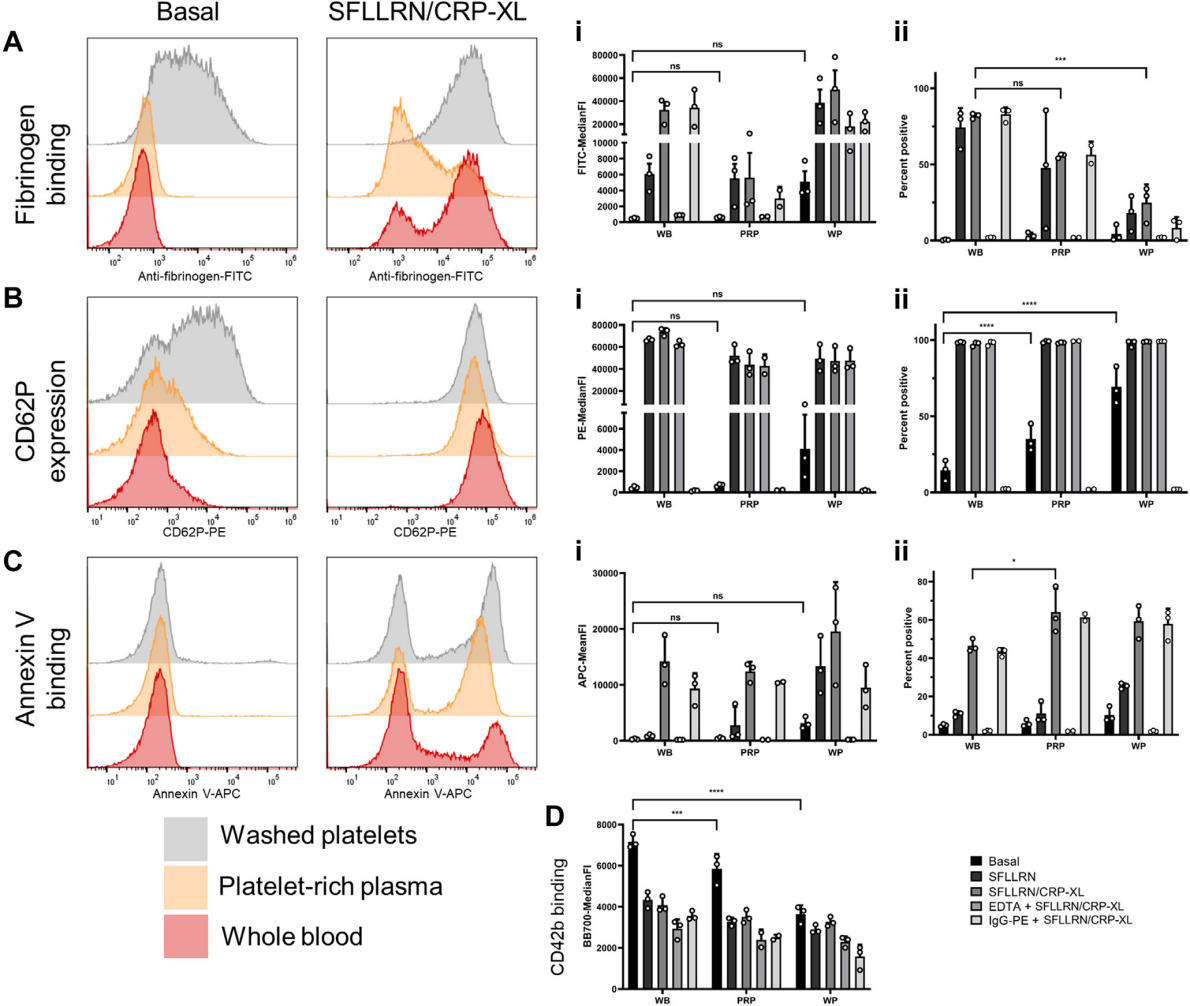
The sample material impacts assay sensitivity. Whole blood was compared with platelet rich plasma and washed platelets for multiparameter platelet activation, at basal or stimulated with SFLLRN (20 μM) alone and with CRP-XL (10 μg/mL). Stimulated EDTA and IgG controls are included for reference to assay performance. (A) Fibrinogen binding, (B) CD62P expression, (C) PS exposure, (D) CD42b expression, (i) indicates MFI data and (ii) percentage positive data (*n* = 3 ± SD). MFI, mean/median fluoresence intensities.

Basal fibrinogen binding was similar in all sample types with a nonsignificant increase in WP compared with PRP and WB. However, agonist-stimulated (SFLLRN/CRP-XL) fibrinogen binding was similar between whole blood and WP, but fibrinogen binding was diminished in PRP (Figure 3A). On further examination of the percent positive data, WP also lost reactivity compared with whole blood (*P* < .005). The EDTA-treated control indicates the high MFI in WP is due to an increased background signal, which in turn affects the ability to detect true positive signal (Figure 3A). This may be a result of the increased binding of fibrinogen to the platelets during isolation, which occurs prior to the EDTA addition. In terms of CD62P expression, there was a significant increase in basal expression linked to artifactual activation when measured as percentage. This was high in PRP (*P* < .001, 35.1 ± 9.1%) and higher still in WP (*P* < .001, 69.5 ± 12.2%) compared with whole blood (14.5 ± 6.7%) (Figure 3B). Paired with this, CD62P expression in both isolated samples showed a nonsignificant reduction in agonist (SFLLRN/CRP-XL) driven expression when compared with whole blood (Figure 3B). Annexin V binding at basal was increased in both PRP and WP (Figure 3C), with a significantly greater proportion of platelets positive for the marker when stimulated (Figure 3C). It has been previously shown (in whole blood) that the number of PS positive cells does not change based on agonist availability [8] suggesting the increased PS exposure may be due to increased artefactual preactivation of these cells, driving hypersensitivity to stimulation. CD42b binding data mirrored CD62P expression and showed a loss of signal in basal samples, mimicking the increased basal activity, in both PRP (*P* < .005) and WP (*P* < .001) while sensitivity to agonist (SFLLRN/CRP-XL) stimulation was maintained (Figure 3D).

### 3.4 Storage

**Figure 4:**
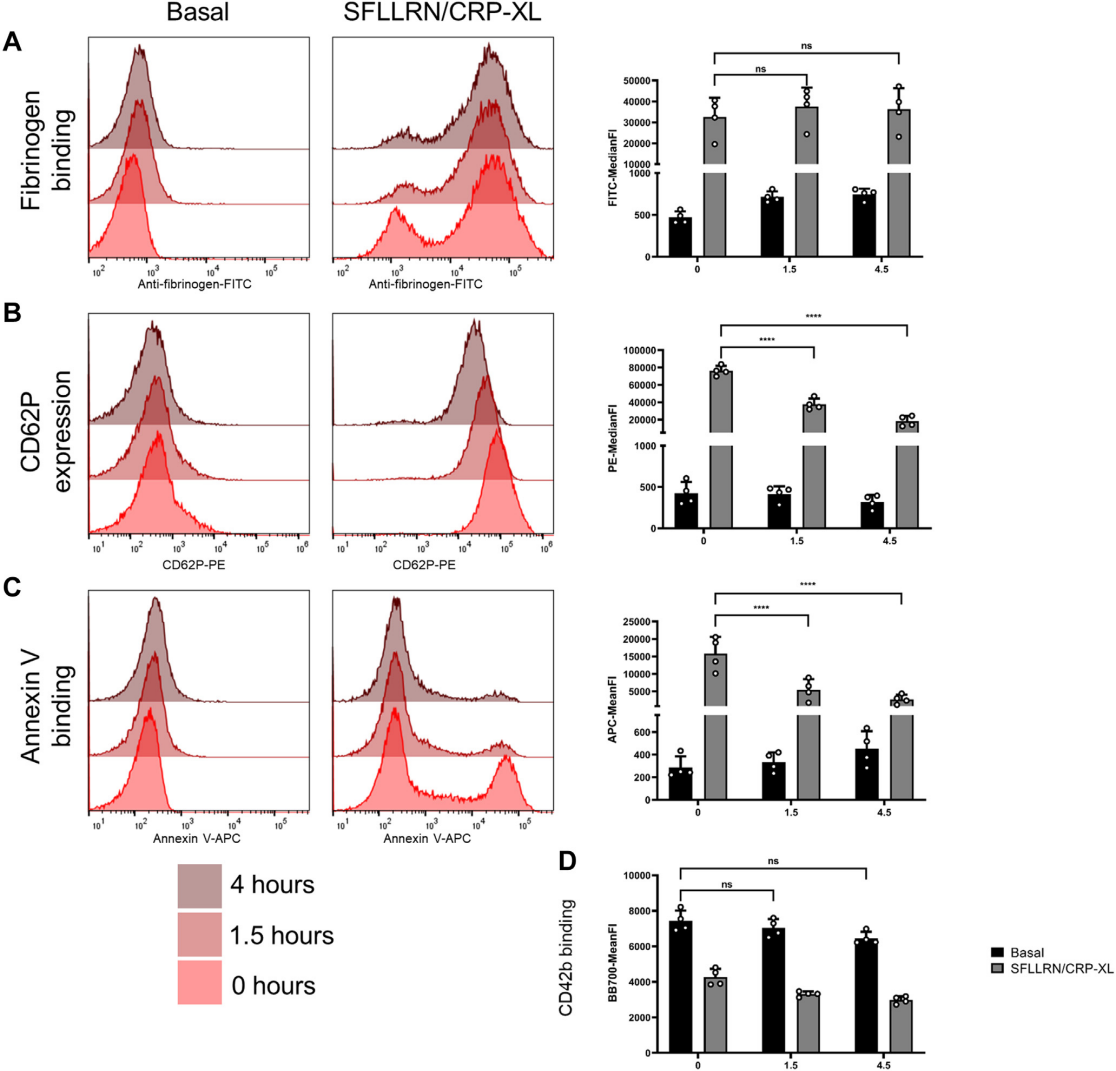
Effect of sample storage on assay performance. Samples fixed and ran immediately compared with 1.5 and 4.5 hours at 4 °C dark storage were compared for multiparameter platelet activation, at basal or stimulated with SFLLRN (20 μM) with CRP-XL (10 μg/mL). (A) Fibrinogen binding, (B) CD62P expression, (C) PS exposure, (D) CD42b expression, (i) indicates MFI data and (ii) percentage positive data (*n* = 4 ± SD). MFI, mean/median fluoresence intensities.

Here we stained and acquired citrated whole blood samples immediately after fixation, and then after storage (at 4 °C in the dark) for 1.5 hours and 4.5 hours the samples were repeatedly acquired for a second and then third time accordingly. We found that measures of fibrinogen binding remained robustly stable with no change in MFI (Figure 4A). However, there was a significant reduction (*P* < .001) in detection of agonist-stimulated CD62P expression at both 1.5 and 4.5 hours (Figure 4B) mirrored by a significant reduction (*P* < .001) in detection of agonist-stimulated annexin V binding at 1.5 and 4.5 hours (Figure 4C). CD42b expression was not significantly altered over the storage times tested (Figure 4D).

### 3.5 Multidimensional analysis

**Figure 5:**
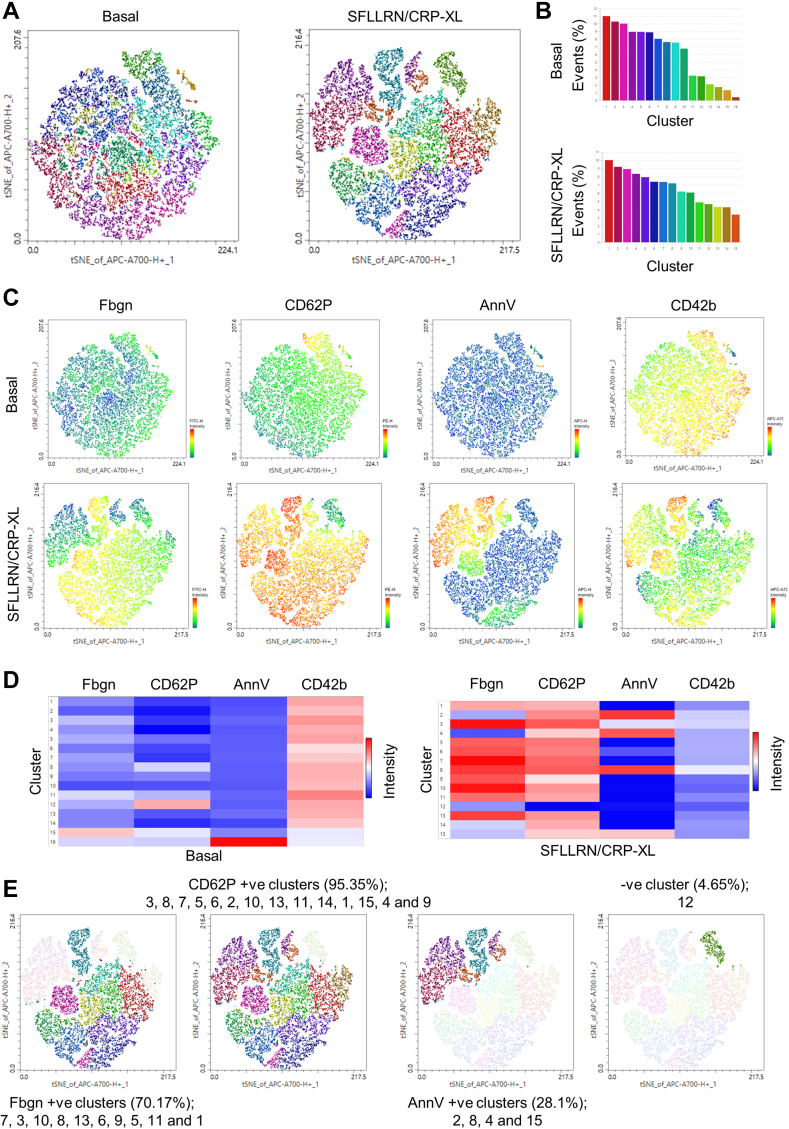
opt-FIt-SNE and Phenograph analysis of platelet subpopulations under optimal preanalytical conditions. 30,000 platelet events from either basal samples or samples treated with SFLLRN (20 μM) and CRP-XL (10 μg/mL) were concatenated and analyzed by opt-FIt-SNE and Phenograph then visualized by ClusterExplorer. (A) tSNE map false colored for Phenograph clusters described in (B). (C) tSNE map false heat-colored (hot-cold/high-low) for indicated marker expression. (D) Heat map (hot-cold/high-low expression) for each cluster identified by Phenograph and relative marker expression within each subpopulation. (E) Stimulated sample only tSNE map highlighted with clusters positive for each marker. tSNE, t-stochastic neighborhood embedded.

Using our optimal conditions described in the previous sections, we performed opt-FIt-SNE [33,34] and Phenograph [35] clustering on concatenated data sets from replicate experiments. Here, we analyzed both basal and stimulated samples, which allow for a direct comparison of the changes to the phenotypic subsets induced by robust dual-agonist (SFLLRN/CRP-XL) driven platelet activation. Combining these 2 techniques allows the projection of 4D data onto a 2D tSNE map (Figure 5A), which is false colored by the Phenograph clusters (Figure 5B). While similar numbers of clusters are identified when comparing basal and stimulated, the subsets are more distinct when the samples have been stimulated. The tSNE map is heat-scaled for expression of each of the 4 parameters and shows both high (warm) and low (cool) zones of expression across the population (Figure 5C). Many of these zones of high and low expression are also identified as distinct subpopulations by Phenograph, which suggests that these tools are clustering and identifying analogous subsets. The clusters identified across the platelet populations by Phenograph can also be compared for relative expression of each marker using a heat map, allowing interrogation of the marker expression profile and proportion of each individual subpopulation (Figure 5D). Together these data demonstrate the almost universally low expression of activation markers on a basal sample, contrasted with the predominantly high but differential expression in stimulated samples (Figure 5D, E). Finally, it is possible to retrospectively project these Phenograph subpopulations onto the tSNE map (Figure 5E), highlighting clusters by their positivity for each individual marker, their unique fingerprint.

## 4 Discussion

In the following sections, we describe our preanalytical, anticoagulant, sample processing time, biological sample material, and sample storage recommendations for development of multiparameter platelet function FFC assays. We present data from samples which were simultaneously probed for 4 markers, and from this, we assess the impact of different conditions on each marker individually. Finally, we investigate the optimum conditions with advanced analytical techniques.

### 4.1 Anticoagulants

Based on our findings, here, we suggest that citrate is the optimal anticoagulant for measuring multiple aspects of platelet function in the same assay sample. Citrate gave the greatest sensitivity to stimulation, reflected in the highest level of agonist-induced fibrinogen binding and the lowest level of basal activity demonstrated by CD62P expression compared with the other anticoagulants tested. Furthermore, citrate did not impede the ability to detect procoagulant platelets, while staining of CD42b was consistent with other anticoagulants. Our findings also corroborate the observations made by others, while providing additional information by measuring markers of platelet function which have not been previously characterized in this way. Citrate, heparin and hirudin anticoagulants were previously assessed for their effects on PAC1 (activated integrin αIIbβ3), CD62P, and LAMP1 [41,42] and in agreement with our study, citrate tubes were considered optimal. Another study comparing citrate, EDTA, heparin, and several coagulation factor inhibitors measuring only CD62P identified that citrate allowed a greater degree of platelet activation [43]. Additionally, EDTA and heparin can drive cell swelling and artifactual activation [32,41,44], and EDTA maintains platelets in a state of hypo-reactivity [45]. The potent inhibitory effects of EDTA are likely driven by the high-binding constant of metal ions when compared to a milder chelator like citrate [46,47], where metal ions, in particular calcium, are vital for platelet activation [48]. In lieu of the availability of citrate anticoagulated blood, heparin anticoagulated would be suggested as the second-line choice.

### 4.2 Sample processing

During storage after blood collection, platelets begin to lose reactivity and become basally activated [32]. This challenge has led to a stringent set of conditions for storage of isolated platelets in a clinical setting. While these conditions can allow for several days of storage, even under these optimal conditions reactivity wanes [39,41] and the platelet storage lesion develops [49]. We believe that the time-frames used here mimic delays which could be experienced by platelet researchers waiting for receipt of samples.

The significant fall in fibrinogen binding did not directly mirror the smaller reductions observed in CD62P and CD42b expression. This may suggest a compounding factor beyond a loss of platelet reactivity during storage is involved. Work from others suggests loss or inactivity of integrin α_IIb_β3 is unlikely to account for this as PAC1 binding does not decrease over time [50] nor does fibrinogen degrade during storage at ambient temperature [51]. GPRP addition is a possible reason for the loss of fibrinogen binding, as it may be incompatible with samples that are stored for extended times [52]. As an alternative to fibrinogen binding, PAC1 may be used to detect the activation of integrin αIIbβ3 [53]. While the use of PAC1 may alleviate loss of fibrinogen signal, there would still be a time dependent decrease in reactivity, as demonstrated by other markers measured here. While it is currently considered appropriate to rest samples for up to 30 minutes before *ex vivo* activation [16], we would recommend that sample preparation occurs as soon as possible after venipuncture. These data suggest there is increased basal activity paired with diminished reactivity as storage time increases. Where delays in sample processing are unavoidable, they should be standardized and increases in basal platelet activity paired with diminished reactivity should be anticipated and appreciated.

### 4.3 Biological samples

Platelet flow cytometry is commonly performed in whole blood samples, but PRP and WP can also be used. Sample material appears to have a significant impact on platelet reactivity and/or increases in basal expression. It is the process of isolation, manipulation, centrifugation, time, and buffers required to isolate platelets from the sample which likely affects platelet function.

The sample material is an important choice when designing experimental protocols for primary cells. Our data here suggest that for analysis of platelet function, whole blood is optimal as it allows the greatest response to agonist stimulation and the lowest levels of artifactual activation. The use of isolated platelets in the forms of PRP or WP led to increased basal activation of markers, likely a combinatory effect of isolation time, physical manipulation (pipette and centrifuge), and buffer changes. However, as we previously examined the effects of time alone, which is different to data presented here, this suggests that manipulation and buffer changes play the main role in affecting platelet function compared with time alone. The significant increase in basal activity means that percent positive cells loses utility, as basal signal may no longer truly represent the activation state of platelets *in vivo.* This is an issue where clinical models are used, as changes in basal activity are often a key observation. However, the use of PRP or WP to measure *in vitro* sensitivity to agonists/inhibitors remains a viable option, or indeed a necessity in instances where reagents may not be compatible with whole blood. Ultimately where high-sensitivity assays are required to comment on basal activation status of platelets, particularly in clinical samples, or to identify platelet subpopulations, whole blood should be the biological sample of choice.

### 4.4 Storage

Stable storage of prepared samples is an important consideration in flow cytometry assays, particularly in settings with core facilities where machine load determines availability. Having determined previously that citrated whole blood is the optimal approach for these experiments we focused on this particular condition to understand the effects of short-term fixed sample storage.

While fibrinogen binding and CD42b remained robust, CD62P expression and annexin V binding were diminished by storage. Other studies have shown that as a marker CD62P is stable up to 5 days [54]; however, this is highly dependent on both the fluorophore-conjugate [55] and storage buffer, as formaldehyde can induce fluorophore quenching [56]. For longer term storage, it may become prudent to wash the cells out of fixative and resuspend in phosphate-buffered saline. However, it is important to note that the decrease in MFI is not reflected by a decrease in the number of cells staining positive for these markers (data not shown), meaning quantitative value is retained in these samples. We recommend that samples be acquired as soon as possible, unless a standardized storage time is used to minimize errors among data that are acquired at different time points.

### 4.5 Multidimensional analysis

Software for multidimensional analysis are being increasingly used in the field of platelet biology, with multiple functions now recognized beyond conventional hemostasis and thrombosis [57]. Opt-FIt-SNE is a method of dimensionality reduction, where events with similar characteristics are plotted in 2-dimensional (2D) space for robust and semi-automated visualization of subsets of related cells. Phenograph clusters cells based on shared characteristics and identifies subpopulations with common phenotypic signatures. Performing the 2 analyses together enriches the discovery of any subsets within the data.

In this example, most cells (95.35%) are CD62P positive, whereas only a small number of cells (4.65%) are low for expression of all markers. The data are more interesting when comparing fibrinogen positive (70.17%) and PS positive (28.1%) cells, as they are mutually exclusive in clusters 2, 4, and 15, but are both expressed in cluster 8, which reinforces recent observations by ourselves and others [6,10,58,59]. Ultimately, this is a limited example of both the analytical tools and applications available, but as multiparameter FFC panels develop in complexity the data generated by these methods will become exponentially richer and provide greater novelty and discovery.

## 5 Summary

We have compared several preanalytical factors relevant to multiparameter platelet flow cytometry using a panel of markers which cover several aspects of platelet function (Supplementary Table S2). In the context of this marker-fluorophore combination, the recommendation is that blood is drawn into tubes anticoagulated with citrate, used with minimal manipulation, and that samples are prepared and acquired immediately, unless these steps can be standardized (Table). If data are obtained with high confidence, it can then be examined by advanced analytical tools and used to explore and discover novel platelet phenotypes and subpopulations.

**Table:** Summary of recommended preanalytical conditions. Having compared the effects of anticoagulants, sample processing conditions, biological sample material, and sample storage on platelet function, we have described the optimal condition for platelet flow cytometry for each of these situations.

Recommended conditions	
Preanalytical condition	Optimal condition
Optimal anticoagulant	Sodium citrate
Sample processing	Process as soon as possible
Biological sample	Whole blood
Sample storage	Avoid or standardize storage

## Figures and Tables

**Supplementary Table 1:**
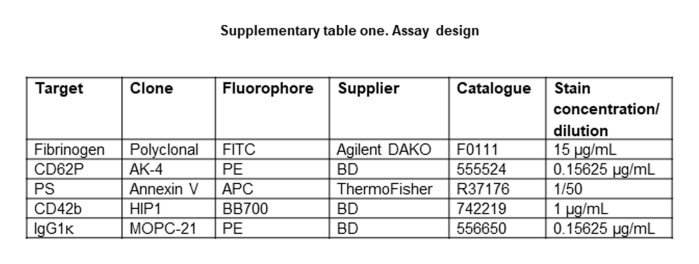


**Supplementary Table 2:**