# Supplementary material for: Preanalytical conditions for multiparameter platelet flow cytometry
Source: Res Pract Thromb Haemost. 2023 Sep 20;7(7):102205. doi: 10.1016/j.rpth.2023.102205 (PMC10579537; doi:10.1016/j.rpth.2023.102205)
Supplement: Supplementary material [file mmc1.docx]

**SUPPLEMENTARY INFORMATION**

**Pre-analytical conditions for multiparameter platelet flow cytometry.**

Matthew S Hindle^1,2^ PhD, Lih T Cheah^1^ PhD, Daisie M Yates^1^ BSc, Khalid M Naseem^1^ PhD

1, Discovery and Translational Science Department, Leeds Institute of Cardiovascular & Metabolic Medicine, University of Leeds, UK.

2, Centre for Biomedical Science Research, School of Health, Leeds Beckett University, UK.

**SUPPLEMENTARY MATERIALS AND METHODS**

**Materials.** Annexin V APC Ready Flow Conjugate (R37176) was from ThermoFisher Scientific. BB700 Mouse Anti-Human CD42b (742219), PE Mouse Anti-Human CD62P (555524), PE Mouse IgG1κ Isotype Control (556650), BD Vacutainer Sodium Citrate (3.2%) Tubes (367691), BD Vacutainer Sodium Heparin (17 U/mL) Tubes (367876), and BD Vacutainer ACD-A (sodium citrate 22.0 g/L, dextrose 24.5 g/L, citric acid 8.0 g/L, K sorbate 0.15 g/L) Tubes (366645) were from BD Biosciences. APC/Cyanine7 Mouse Anti-Human CD42b (HIP1), BV510 Mouse Anti-Human CD41 (HIP8), PerCP/Cyanine5.5 Mouse Anti-Human CD36 (5-271), APC-Cyanine7 Mouse IgG1κ Isotype Control, BV510 Mouse IgG1κ Isotype Control, and PerCP/Cyanine5.5 Mouse IgG2aκ Isotype Control were from Biolegend. Anti-Human Fibrinogen/FITC (F0111) was from Agilent Technologies. Greiner Bio-One Vacuette Potassium EDTA (K_2_EDTA 1.8 mg/mL) (456023) was from Greiner Bio-One. SFLLRN (Thrombin Receptor Activating Peptide) (58927) was from Anaspec. Cross-linked collagen-related peptide (CRP-XL) was from Collagen Toolkits. Prostaglandin I_2_ (sodium salt) (61849-14-7) was from Cayman Chemical. VersaComp Antibody Capture Bead Kit (B22804) was from Beckman Coulter Life Sciences. 4% Paraformaldehyde Aqueous Solution (157-4) was from Electron Microscopy Sciences. All other reagents where not specified were from Sigma-Aldrich.

**Venepuncture.** Blood was drawn from healthy adults with informed consent. Donors were recruited from a pool of healthy unfasted volunteers, who confirmed they had not recently taken drugs which may affect platelet function, blood was taken in the morning. Venepuncture was performed with a 21G butterfly needle into vacutainers ^1^, and the first tube was discarded to minimise artefactual activation. For direct comparison each replicate of anticoagulant vacutainers, PRP and washed platelets were each prepared on the same day from a single donor. All human work was approved by the University of Leeds Medical Ethics Committee.

**Platelet isolation.** For washed platelet (WP) isolation, blood was drawn into 8.5 mL ACD-A vacutainers. Two vacutainers were pooled (17 mL) and centrifuged at 100*g* for 20 min to isolate platelet-rich plasma (PRP). PRP was removed and treated with PGI_2_ (200 nM) ^2^, and then centrifuged at 1000*g* for 10 min to pellet platelets. The platelet pellet was resuspended in a wash buffer of 90% (v/v) modified Tyrode’s buffer (0.5 mM MgCl_2_, 0.55 mM NaH_2_PO_4_, 2.7 mM KCl, 5 mM HEPES, 5.6 mM glucose, 7 mM NaHCO_3_, 150 mM NaCl, pH 7.4) and 10% (v/v) ACD (2.9 mM citric acid, 29.9 mM sodium citrate, 72.6 mM NaCl, 113.8 mM glucose, pH 6.4) then treated with PGI_2_ (200 nM) and centrifuged at 1000*g* for 10 min to pellet platelets. The final pellet was resuspended in pre-warmed (37^o^C) modified Tyrode’s buffer and counted using a Beckman Coulter Counter Z1 and adjusted to 5x10^8^ platelets/mL. Platelet-rich plasma (PRP) was isolated from sodium citrate vacutainers by centrifugation, 100 *g* for 10 min.

**Supplementary CD42b flow cytometry.** Samples were prepared and analysed as described in the main text but were run on a Beckman Coulter CytoFLEX S with four lasers (of which 405, 488 and 638 nm were used) and 11 detectors (of which 525/40, 690/50 and 780/60 BP filters were used).

**Tetramethylrhodamine ethyl ester (TMRE).** Washed platelets (2x10^6^/tube) were incubated with TMRE (200 nM) for 20 min before dilution in 10x volume of PBS and immediately analysed. As an internal control, these platelets were first incubated for 10 min with carbonyl cyanide 4-(trifluoromethoxy)phenylhydrazone (FCCP, 20 µM) to induce mitochondrial membrane (ΔmΨ) depolarisation. Platelets were gated on SSC/FSC and 10,000 events were recorded, TMRE was excited by the 488 nm laser and fluorescence collected in the 585/42BP filter.

**Supplementary references.**

1. Welch EL, Crooks MG, Hart SP. Agreement between blood draw techniques for assessing platelet activation by flow cytometry. Platelets. 2018: 30:4, 530-534.

2. Vargas JR, Radomski M, Moncada S. The Use Of Prostacyclin In The Separation From Plasma And Washing Of Human-Platelets. Prostaglandins. 1982;23(6):929-945.

**SUPPLEMENTARY FIGURE & TABLE LEGENDS**

**Supplementary figure 1. Platelet gating strategy.** Platelets were gated on their typical physical characteristics on an SSC/FSC dot plot which excluded the majority of red and white blood cells. This population was then sub-gated for positive expression on an SSC/CD42b dot plot which excludes non-platelet debris and doublet events.

**Supplementary figure 2. CD42b as a platelet marker.** Whole blood was stained with CD42b-APC/Cy7 (3 µg/mL) and (A) CD41-BV510 (1 µg/mL) or (B) CD36-PerCP/Cy5.5 (1 µg/mL) followed by fixation and flow cytometric analysis. Platelets were gated on their typical physical characteristics on an SSC/FSC dot plot followed by doublet exclusion. They were then plotted on a CD42b (X-axis) and (A) CD41 or (B) CD36 (Y-axis) scatter plot using matched isotype controls to determine background binding. These experiments confirm CD42b is constitutively expressed on the surface of all CD41/CD36 positive platelets. Representative of n=3.

**Supplementary figure 3. Time-dependent loss of ΔmΨ.** Washed platelets stored at 37^o^C for the indicated times were stained with TMRE (200 nM, 20 min), with or without pre-treatment with FCCP (20 µM, 10 min). n=1.

**Table S1. Assay probe characteristics.** Antibody target, clone, fluorophore and working concentration used within this study.

**Table S2. Assay targets.** List of targets from the four-parameter assay and the component of platelet biology which they represent.
